# All-in-One Self-Powered Human–Machine Interaction System for Wireless Remote Telemetry and Control of Intelligent Cars

**DOI:** 10.3390/nano11102711

**Published:** 2021-10-14

**Authors:** Tingting Zhang, Lingjie Xie, Junyan Li, Zheguan Huang, Hao Lei, Yina Liu, Zhen Wen, Yonglin Xie, Xuhui Sun

**Affiliations:** 1 School of Nano Technology and Nano Bionics, University of Science and Technology of China, Hefei 230026, China; ttzhang2017@sinano.ac.cn (T.Z.); zghuang2017@sinano.ac.cn (Z.H.); 2 Inkjet Printing Technology Research Center, Printable Electronics Research Center, Suzhou Institute of Nano-Tech and Nano-Bionics, Chinese Academy of Sciences, Suzhou 215123, China; 3 School of Science, Xi’an Jiaotong-Liverpool University, Suzhou 215123, China; 20174214077@stu.suda.edu.cn (L.X.); 20124def10@gmail.com (J.L.); Yina.Liu@xjtlu.edu.cn (Y.L.); 4 Jiangsu Key Laboratory for Carbon-Based Functional Materials and Devices, Institute of Functional Nano and Soft Materials (FUNSOM), Soochow University, Suzhou 215123, China; jacklei_zjou@163.com

**Keywords:** human–machine interaction, triboelectric nanogenerator, self-powered sensing, self-charging power unit, remote telemetry and control

## Abstract

The components in traditional human–machine interaction (HMI) systems are relatively independent, distributed and low-integrated, and the wearing experience is poor when the system adopts wearable electronics for intelligent control. The continuous and stable operation of every part always poses challenges for energy supply. In this work, a triboelectric technology-based all-in-one self-powered HMI system for wireless remote telemetry and the control of intelligent cars is proposed. The dual-network crosslinking hydrogel was synthesized and wrapped with functional layers to fabricate a stretchable fibrous triboelectric nanogenerator (SF-TENG) and a supercapacitor (SF-SC), respectively. A self-charging power unit containing woven SF-TENGs, SF-SCs, and a power management circuit was exploited to harvest mechanical energy from the human body and provided power for the whole system. A smart glove designed with five SF-TENGs on the dorsum of five fingers acts as a gesture sensor to generate signal permutations. The signals were processed by the microcontroller and then wirelessly transmitted to the intelligent car for remote telemetry and control. This work is of paramount potential for the application of various terminal devices in self-powered HMI systems with high integration for wearable electronics.

## 1. Introduction

Human–machine interaction (HMI) systems have attracted tremendous attention with the rapid development of information technology and the urgent demands of the internet of things (IoTs) [1,2]. To effectively realize the function of HMI systems, they should traditionally consist of three components: multiblock sensors for detecting or monitoring objects’ signals, microcontroller (MCU) modules for signal reception, identification, and processing, and terminal devices to receive the processed signals and respond accordingly [3,4,5]. Moreover, the energy supply is inevitable to power the system for continuous operation. Current commercialized HMI systems exist in every field that participates in human–machine information exchange, especially as wearable electronics for intelligent control [6,7].

Although there has been a significant advancement in terms of traditional HMI systems, two bottlenecks still need to be broken through to obtain a better wearing experience when the system adopts wearable electronics. First, the three typical components in addition to the energy supply part of HMI systems are independent and dispersed, which presents low integration and difficulty obtaining a comfortable wearing experience [8,9,10]. In addition, continuous energy supply is necessary for powering the sensors and MCU circuits considering the front-to-back structure design of current systems [11,12,13,14]. Fiber energy devices are currently developed for powering wearable systems. Batteries with high densities are commonly used, but are too difficult to make, especially stretchable or fibrous batteries [15,16]. Therefore, the development of HMI systems with high integration and self-powered characteristics is still an intriguing challenge and remains an open area for investigations.

Triboelectric technology has been positively exploited in many fields since Wang’s group first invented triboelectric nanogenerators (TENG), such as self-powered sensors, mechanical energy or blue energy harvesting, and high-voltage occasions [17,18,19,20,21,22,23]. Apparently, the employment of self-powered sensors in HMI systems can effectively reduce energy requirements since they generate electrical signals directly compared to previous resistive or capacitive sensors [24,25,26,27]. Simultaneously, the energy collected by TENG can be temporarily stored in energy storage devices, such as supercapacitors, which has been achieved through self-charging power units (SCPU) [28,29,30,31]. Based on these two functions, triboelectric technology provides a preeminent idea for solving the energy supply and integration problems of traditional HMI systems, which favorably promotes its creation and implementation [32,33].

In this work, we propose a prototype of an all-in-one self-powered HMI system relying on triboelectric technology. The detailed working flow chart of the system is shown in Supplementary Figure S1. First, SF-TENG was fabricated by employing a dual-network crosslinked hydrogel as the electrode and silicone rubber as a coating layer that served as a self-powered sensor. A smart glove was then designed by attaching five SF-TENG sensors on the dorsum of five fingers, which generated signals under different gestures. Then, the smart gloves were connected to the MCU part ulteriorly to detect and distinguish these signals. Supercapacitors with sandwiched structures were designed by attaching the CNT electrode film to the pre-stretched dual-network crosslinked hydrogel electrolytes. SCPU integrated with woven SF-TENGs, series supercapacitors, and a power management circuit were exploited to harvest mechanical energy from the human body and power the systems. Ultimately, the proposed integrated system was successfully applied in wireless remote telemetry and control for intelligent cars, which is of paramount potential for self-powered HMI systems with high integration for wearable electronics.

## 2. Experimental Section

### 2.1. Materials

Sodium alginate (SA), acrylamide (AAm), sodium chloride (NaCl), and calcium sulfate dihydrate (CaSO_4_·2H_2_O) were obtained from Sinopharm Chemical Reagent Co., Ltd. (Shanghai, China). Ammonium persulfate (APS), N, N, N, N’-tetramethylenediamine (TMEDA), and N, N-methylenebisacrylamide (MBAA) were all derived from Sigma-Aldrich (Shanghai) Trading Co., Ltd. (Shanghai, China). Silicone rubber (Ecoflex 00-10) was manufactured by Smooth-On, Inc. (Marco Key, PA, USA). All materials or chemicals above were directly used as received without any further purification. All aqueous solutions were prepared using ultrapure water with resistivity ≥ 18.2 MΩ·cm.

### 2.2. Synthesis of the Hydrogels

AAm powders of 14 g were first dissolved in 60 mL ultrapure water. After 5 min of stirring, 2.8 g SA was slowly added into the solution and stirred for 8 h until completely dissolved. Then, we added different contents of NaCl as the electrolyte. We kept stirring for 30 min and then added MBAA (0.06% the weight of AAm) as the crosslinker and TEMED (0.25% the weight of AAm) as the crosslinking accelerator for polyacrylamide (PAAm), respectively. Then, APS (0.5% the weight of AAm), which was completely dissolved in 20 mL ultrapure water, was added as the initiator of PAAm. CaSO_4_·2H_2_O (13% the weight of SA) as the ionic crosslinker for SA was dissolved in 20 mL ultrapure water and added dropwise to the pre-mixed solution. We then poured the gel solution into a PTFE mold with a size of 20 × 20 × 1 mm^3^ and put it into a 50 °C oven for about three hours until the hydrogel was cured.

### 2.3. Fabrication of Fibrous and Woven TENG

The silicone rubber was used by mixing components A and B in a 1:1 weight ratio. The fibrous TENG was fabricated by coating the silicone rubber on the stretchable hydrogel electrode. As the shape of the hydrogel electrode was strip-shaped, after the silicone rubber was cured, the fibrous TENG was close to a cylindrical shape. The TENG fabric was obtained by manual weaving.

### 2.4. Fabrication of Supercapacitor

The hydrogel was first stretched to a strain of 400% and fixed with two clips; then, we carefully attached the CNT film on both sides to ensure that the electrodes did not touch each other and cause a short circuit. When multiple supercapacitors are connected in series, the upper and lower electrodes alternately replace external wires to form the integrated supercapacitor. After releasing the stain, we coated the silicone rubber on the outside as the encapsulation layer. Finally, we connected the CNT electrodes of the supercapacitors to the output port of the power management circuit by conductive tape for storing the energy from the TENG.

### 2.5. Characterization and Measurement

A scanning electron microscope (QUANTA FEG 250) (FEI Company, Hillsboro, OR, USA) was employed to characterize the surface morphology of the hydrogel. The Instron 3366 electronic universal testing machine (Instron Corporation, Canton, MA, USA) was employed to test the mechanical tensile strength of the hydrogel. The electrical output performance of the TENG, including short-circuit current, open-circuit voltage, and transfer charge, was tested by a programmable electrometer Keithley model 6514 ( Tektronix, Beaverton, OR, USA), and real-time data acquisition was realized by a software platform, which was constructed based on the LabView (LabView 2015, National Instruments, Austin, TX, USA). For the supercapacitor electrochemical performance, such as cyclic voltammetry (CV) and galvanostatic charge–discharge (GCD) measurement, the electrochemical workstation (CHI 760E, Shanghai Shinstruments Co., Ltd., Shanghai, China) was utilized. The electrochemical impedance spectroscopy (EIS) was also tested by the electrochemical workstation. A piece of hydrogel with a size of 1 × 1 cm^2^ was sandwiched between two sheets of stainless steel. The ionic conductivity was calculated by *σ* = *d*/(*R* × *A*), where *d* and *A* denote the thickness and area of the sample, respectively, and *R* is determined by the intercept of the EIS real axis.

## 3. Results and Discussion

### 3.1. Synthesis and Characterizations of Materials

**Figure 1:**
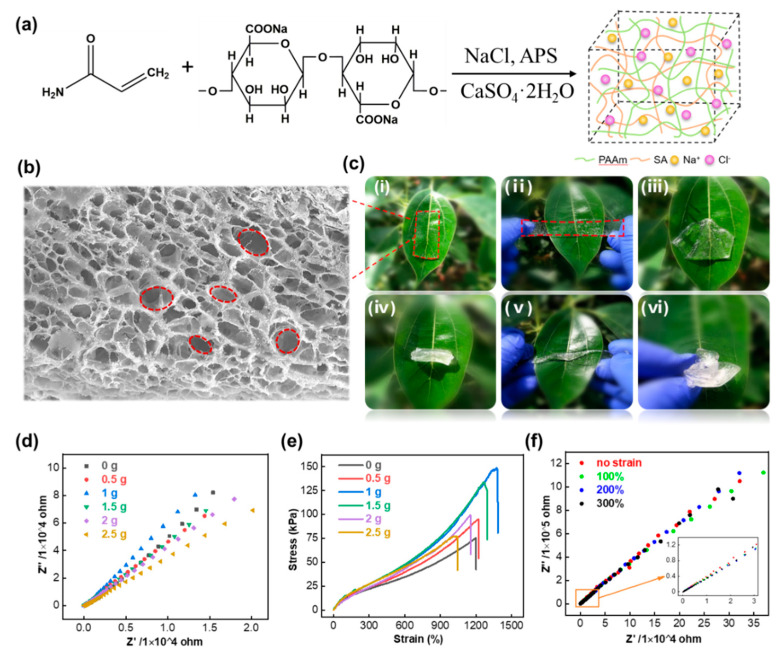
Synthesis and characterizations of dual-network crosslinked hydrogels. (**a**) Synthetic route of hybrid hydrogels containing sodium alginate and polyacrylamide with CaSO_4_·2H_2_O and APS served as initiators. (**b**) SEM image of freeze-dried composite hydrogels (scale bar: 50 μm). (**c**) Photographs of the hydrogel at different deformed states, namely, (ⅰ) original, (ⅱ) stretching, (ⅲ) rolling, (ⅳ) folding, (ⅴ) twisting, and (ⅵ) crumpling, respectively. (**d**) Electrochemical impedance spectroscopies (EIS) and (**e**) typical tensile stress–strain curves of hydrogel with different NaCl contents. (**f**) EIS of hydrogels with different strains when the content of NaCl was fixed at 1.5 g.

The desired stretchable hydrogels were facilely synthesized by blending two monomers, namely, a covalently crosslinked polymer, polyacrylamide, and an ionically crosslinked polymer, alginate, followed by adding the initiator APS and CaSO_4_·2H_2_O, respectively [34]. By dual-network crosslinking, the hydrogels obtained both elasticity and toughness, as shown in Figure 1a. The obtained hydrogel had three-dimensional hollow network structures from the scanning electron microscopy (SEM) image (Figure 1b). FTIR spectroscopy was also measured to further confirm the composition of the hydrogel (Supplementary Figure S2). Besides the high transparency and high stretchability (Supplementary Figure S3), the hydrogel also endured multiple deformation forms, including rolling, folding, twisting, and crumpling (Figure 1c). To enhance the ionic conductivity, different weight contents of NaCl between 0 and 2.5 g (about 17.8% the weight of AAm) were added into the hydrogel solution, and the electrochemical impedance spectroscopy (EIS) (Figure 1d) was measured. As shown in Supplementary Figure S4, the maximal ionic conductivity calculated from the EIS was 19.86 mS/cm when the NaCl content was 1.5 g, which was 14 times higher than the hydrogel without NaCl. This enhancement is mainly due to the increased abundance of free ions. As seen in Figure 1e, the addition of NaCl not only improved the ionic conductivity but also enhanced the mechanical property of the hydrogel. The stress reached more than 1300% when the NaCl content was 1.5 g. This is mainly attributed to the salting-out effect of NaCl, which induced the PAAm chain entanglements or formed microcrystalline zones, further strengthening the hydrogel’s mechanical performance. Moreover, the ionic conductivity almost stayed the same when stretching the hydrogel (Figure 1f), which ensured the stability to fabricate functional devices. Given these two key focuses of mechanical properties and ionic conductivity, the hydrogel synthesized by adding 1.5 g NaCl was employed for device fabrication in the next experiment.

### 3.2. Electrical Performance of the SF-TENG

**Figure 2:**
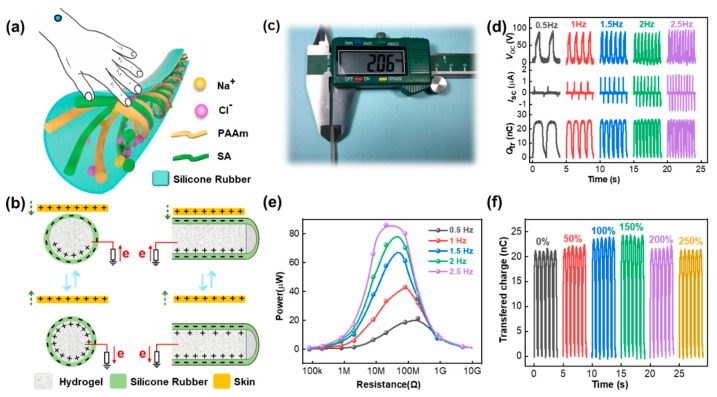
Working mechanism and electrical performance of the stretchable fibrous triboelectric nanogenerator (SF-TENG) at singleelectrode mode. (**a**) Schematic illustration of the SF-TENG when in contact with skin. (**b**) The working mechanism of the SFTENG for generating electricity under shortcircuit conditions. (**c**) Photograph of diameter measurement of TENG. (**d**) The electrical output performance of the single fiberbased TENG with different contact frequencies ranging from 0.5 to 2.5 Hz, including *V*_oc_, *I*_sc_, and *Q*_tr_. (**e**) Dependence of the output power under external resistance load with different frequencies from 0.5 to 2.5 Hz. (**f**) Transferred charges under various strains (0~250%).

A fibrous stretchable TENG (FS-TENG) at the single-electrode mode was fabricated by coating the silicone rubber outside of the hydrogel ionic conductor, as shown in Figure 2a. The reason why silicone rubber was chosen as the encapsulation layer is due to its good stretchability, strong electron affinity and ability to prevent the hydrogel from losing water. Figure 2b schematically illustrates the working mechanism of SF-TENG for generating electricity under short-circuit conditions. The left part is from the front view, and the right part is a corresponding side view with a half-cut. When skin contacted the SF-TENG, positive and negative charges were generated on the surface of the human skin and silicone rubber, respectively, due to their different electron affinities. Once the skin was separated from it, the interaction between the two opposite polarity charges decreased, leading to positive charges induced on the ionic conductor and electrons transferred to the outside from connected wires. When skin contacted the SF-TENG again, the triboelectric charges also returned to their original distribution state. The diameter of the SF-TENG is about 2 mm (Figure 2c), which is suitable for wearable devices. Simultaneously, it has excellent stretchability, which reaches 300% without fracture or breakage, as illustrated in Supplementary Figure S5. To evaluate the electrical output performance of the SF-TENG, the open-circuit voltage (*V*_oc_), short-circuit current (*I*_sc_), and transferred charges (*Q*_tr_) were measured periodically by the linear motor. The testing schematic diagram of TENG at original and stretching states is provided in Supplementary Figure S6. The human skin was replaced by hog skin for long-term testing, and the area of hog skin was much larger than the TENG surface to ensure full contact between them. The electrical output of 10 cm TENG with the frequency varying from 0.5 to 2.5 Hz is shown in Figure 2d. It is noted that the *V*_oc_ and *Q*_tr_ almost stayed the same with values of 89.7 V and 26.3 nC, respectively. The *I*_sc_ increased from 0.53 to 1.58 μA with the increasing frequency. Moreover, the optimal resistance load decreased gradually with an increase in frequency, which is demonstrated in Figure 2e. This is mainly attributed to the decrease in intrinsic resistance of TENG with a higher movement speed [35]. Furthermore, the power reached the maximum value (~86.1 μW) at 20 MΩ when the frequency was 2.5 Hz. The transferred charges under different strains (0–250%) are shown in Figure 2f. It can be observed that as the strain increased, the *Q*_tr_ increased gradually until the strain reached 150% and then decreased. As displayed in Supplementary Figure S7, two factors caused this tendency: one was the surface charge enhancement due to the thinner thickness of silicone rubber, which had a positive effect on the output, and the other was the resistance enhancement of hydrogel electrode, which has the reverse effect. The former was dominant in small strains (<150%), while the latter had a greater impact when the strain was larger.

### 3.3. Fabrication Process and Electrochemical Performance of SF-SC

**Figure 3:**
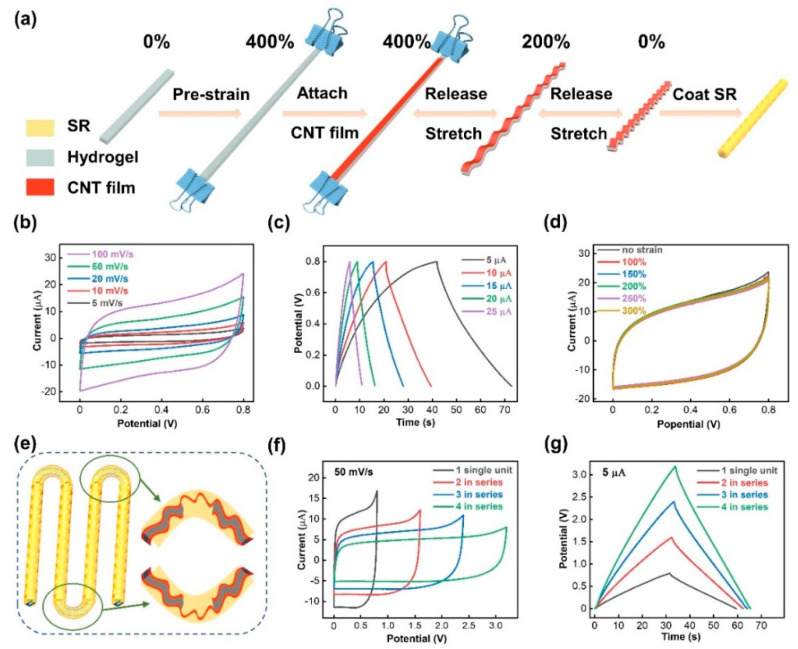
Schematic illustration and electrochemical performance of the stretchable fibrous supercapacitor (SF-SC). (**a**) Schematic illustration of the fabrication process. (**b**) CV curves of the SF-SC at different scanning rates (5–100 mV/s). (**c**) GCD curves at different current loadings (5–25 μA). (**d)** CV curves of the SF-SC under different strains (0–300%). (**e**) Schematic illustration of the series-connected SF-SCs. (**f**) CV and (**g**) GCD curves of various numbered series-connected SF-SCs (1 to 4).

The hydrogel was also exploited as the electrolyte of the stretchable fibrous supercapacitor (SF-SC) as a common ionic conductor. To endow the supercapacitor with stretchability, CNT film was attached on both sides of the pre-stretched hydrogel, as illustrated in Figure 3a. The performance of SF-SC was characterized by the CV and GCD techniques provided by the electrochemical workstation. As shown in Figure 3b, the CV curves of a single unit SC were close to the rectangular shape at different scan rates ranging from 5 to 100 mV/s with the selected voltage window (0 to 0.8 V), thus indicating the quick electrochemical switching ability and good reaction reversibility of the device. The GCD curves with a typical symmetric triangular shape under the current load of 5 to 25 μA are shown in Figure 3c, which further validates the excellent capacitive behavior of the SC. The capacitance calculated from the GCD curves by the equation *C* = (*i* × Δ*t*)/Δ*V* was about 156.3 μF when the current load was 5 μA [36]. Figure 3d shows the CV curves of SF-SC at different strains, and there was little capacitance drop observed under each tensile state condition. For the sake of practical applications of the SF-SC as an energy supply, several single unit SCs were connected in series. Unlike the traditional wire connection methods, which are fragile and unable to endure large stains, the series SF-SCs were connected by the CNT film itself inside and silicone rubber outside to form an all-in-one device (Figure 3e). The CV and GCD curves of 1 to 4 units connected in series are displayed in Figure 3d,f, respectively. It can be observed that the voltage increased linearly according to the number of series-connected SC. Thus, the results demonstrate the fabricated SF-SCs have good adjustability and adaptability to various electronic applications.

### 3.4. Demonstration of the Self-Charging Power Unit

**Figure 4:**
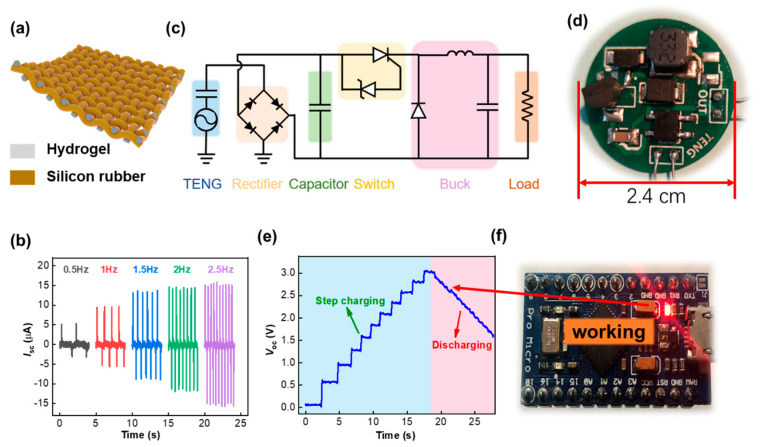
Demonstration of the self-charging power unit. (**a**) Schematic illustration of woven SF-TENGs into cloth. (**b**) Short-circuit current output with different contact frequencies ranging from 0.5 to 2.5 Hz. (**c**) Circuit diagram of the self-charging power unit containing woven SF-TENGs, power management system, and load. (**d**) Photograph of the power management system. (**e**) Charging and working curve of four series-connected SF-SCs by manually patting the woven SF-TENGs cloth and (**f**) powering the microcontroller.

Figure 4 demonstrates the SCPU performance, which consisted of woven SF-TENGs, series-connected SF-SCs, and a power management circuit for high charging efficiency. In light of the pulse and low current output of a single unit SF-TENG, several devices were woven together for the practical application, as shown in Figure 4a. The short-circuit current output of woven SF-TENGs was largely enhanced compared with the single unit; for example, the *I*_sc_ reached 15.8 μA at a contact frequency of 2.5 Hz (Figure 4b). As we know, a typical feature of TENG’s electrical output is high voltage but low current, due to the pulsed output voltage with a short duration and limited frequency, making TENG unsuitable for direct use as a power source. Thus, a power management circuit was designed to convert the pulsed voltage into a steady continuous output (Figure 4c). The power management system mainly contained a rectifier to convert the AC to DC, a fixed value capacitor based on the TENG’s inherent capacitance to extract maximal energy from the power source, a switch containing the silicon-controlled rectifier (SCR) and Zener diode to control the power flow paths, and a buck converter circuit. Figure 4d is the photograph of the power management circuit of which the diameter is about 2.4 cm, which is small enough to treat as a button or decoration in wearable electronics. Figure 4e shows the charging and working curve of four series-connected SF-SCs by manually patting the woven SF-TENGs cloth. It is worth noting that the charging voltage had a step-up characteristic attributed to the switching mode of the power management circuit. The switch did not turn on until the fixed value capacitor was charged to a specified voltage, which was set by the Zener diode. Once the switch turned on, the fixed value capacitor discharged to transfer the energy into the load through the buck converter circuit. After the discharge process was over, the switch turned off again and isolated the fixed value capacitor from the load. Considering that the sensor in the system was not continuously working, but was in a long-term dormant state, the SCPU, which always generated electricity, could trigger the MCU if the sleeping mechanism was included in the program. As shown in Figure 4f and Movie S1, when the series-connected SF-SCs were charged to 3 V in about 18 s, an Arduino Leonardo was successfully powered to work as the power-up indicator LED turned red, which would then be used for signal processing in the all-in-one self-powered HMI system.

### 3.5. Application of All-in-One Self-Powered HMI System

**Figure 5:**
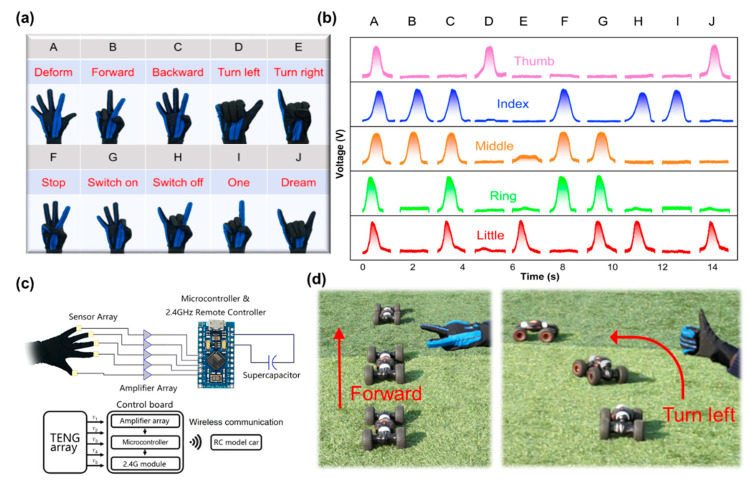
Application of all-in-one self-powered HMI system. (**a**) Photograph of a smart glove with five SF-TENG sensors attached on the dorsum of fingers, and images of ten different sign language gestures. (**b**) Real-time monitored relative voltage changes under various sign language gestures as shown in (**a**). (**c**) Block diagram of the communicator based on SF-TENG sensors. SF-TENG sensors were connected to a control board that contains an amplifier array, a microcontroller, and a 2.4 GHz remote controller. (**d**) Photographs of 2 gestures (top inset), command codes (bottom inset), and corresponding motions of the intelligent car.

A smart glove was developed by attaching five SF-TENG sensors on the dorsum of five fingers (thumb/index/middle/ring/little finger) acting as the self-powered gesture sensors. As each sensor on different fingers may have undergone different squeeze deformations upon various gestures, their corresponding voltage output also differed and, therefore, real-time signal permutations could be achieved. For example, when all five fingers remained straight, the voltage outputs affiliated with them were kept approximately at zero. When one finger repeated the movement of bending and releasing, the sensor on this finger would generate a relatively high pulse voltage compared to the other four motionless fingers. Based on such results, ten different sign language permutations named from A to J are displayed in Figure 5a. The real-time monitored relative voltage changes corresponding to these sign language permutations are shown in Figure 5b. It is noted that different sign language gestures brought about completely different voltage combinations, thus indicating there will be dozens of signals, in theory, by one hand gesture. To detect and distinguish these signals, the smart glove was connected to a control board that contained an amplifier array, a microcontroller, and a 2.4 GHz remote controller. Simultaneously, the series-connected SF-SCs, which were charged to the working voltage by the woven SF-TENGs, provided energy to this control board. The block diagram of the entire system based on triboelectric technology is shown in Figure 5c. The signal was processed and decoded to a specific RC command via a programmable mapping logic system. Supplementary Figure S8 displays the state of the command code. The decoded command would then be wirelessly transmitted to the terminal product. The circuit model for connecting the smart gloves and terminal devices is schematically illustrated in Supplementary Figure S9. Specifically, as shown in Figure 5d and Movie S2, when the gestures with forward and turn left command were executed, the intelligent car received the signals wirelessly sent from the MCU and made a corresponding displacement movement. This demonstration shows the feasibility of adopting all-in-one self-powered HMI systems to achieve highly accurate gesture control for intelligent equipment.

## 4. Conclusions

In conclusion, an all-in-one self-powered human–machine interaction system based on triboelectric technology was successfully fabricated by the combination of a self-powered triboelectric sensor, microcontroller, intelligent car, and self-powered charging unit. Dual-network crosslinking hydrogel with a high stress of 1300% and ion conductivity of 19.86 mS/cm wrapping with the silicone rubber dielectric layer fabricated the SF-TENG. A smart glove with five SF-TENG on the dorsum of five fingers served as self-powered sensors and generated signal permutations with different gestures. The signals were processed by the microcontroller and then wirelessly transmitted to the intelligent car for remote telemetry and control. During the working process, the SCPU integrated by woven TENG, a supercapacitor of 156.3 μF, and a power management circuit was exploited to harvest mechanical energy from the human body and successfully power the systems in about 18 s. Since the sensing and energy harvesting functions are both achieved by TENG, this all-in-one system is compactly integrated. In addition, due to the universality of various terminal devices, the system has great potential application value in self-powered HMI systems with wearable electronics.

## Data Availability

The data presented in this study are available on request from the corresponding author.

## Supplementary Materials

The supplementary materials are provided by the supporting information file. https://www.mdpi.com/article/10.3390/nano11102711/s1. Figure S1: The working flow chart of an all-in-one self-powered human-machine interaction system, Figure S2: FTIR spectra of the freeze-dried composite hydrogel, Figure S3: Optical images of hydrogel with different strains. The content of NaCl was fixed as 1.5 g, Figure S4: The calculated ionic conductivity(σ) of hydrogel with different NaCl contents, Figure S5: (a) Photos of SF-TENG at the original state and stretched state of 300% strain, (b) Diameter change at different stretch rates (0–300%), Figure S6: The testing schematic diagram of TENG at (a) original and (b) stretching state, Figure S7: The surface of silicone rubber becomes thinner (a) and the resistance of hydrogel increase (b) during stretching, Figure S8: The processed and decoded RC command via a programmable mapping logic system, Figure S9: The circuit model for connecting the smart gloves and terminal devices. Video S1: An Arduino Leonardo is successfully powered to work by the SCPU. Video S2: Wireless remote telemetry and control of intelligent car by smart glove.
