# Supplementary material for: All-in-One Self-Powered Human–Machine Interaction System for Wireless Remote Telemetry and Control of Intelligent Cars
Source: Nanomaterials (Basel). 2021 Oct 14;11(10):2711. doi: 10.3390/nano11102711 (PMC8539533; doi:10.3390/nano11102711)
Supplement: Supplementary file 1 [file nanomaterials-11-02711-s001.zip › nanomaterials-1409777 supplementary 10.22 for conversion new.pdf]

# Supplementary Materials

# All-in-One Self-Powered Human–Machine Interaction System for Wireless Remote Telemetry and Control of Intelligent Cars

**Tingting Zhang [1,2], Lingjie Xie [3], Junyan Li [3], Zheguan Huang [1,2], Hao Lei [4], Yina Liu [3], Zhen Wen [4,*], Yonglin Xie [2,*] and Xuhui Sun [4,*]**

1 School of Nano Technology and Nano Bionics, University of Science and Technology of China, Hefei 230026, China; ttzhang2017@sinano.ac.cn (T.Z.); zghuang2017@sinano.ac.cn (Z.H.)
2 Inkjet Printing Technology Research Center, Printable Electronics Research Center, Suzhou Institute of Nano-Tech and Nano-Bionics, Chinese Academy of Sciences, Suzhou 215123, China
3 School of Science, Xi'an Jiaotong-Liverpool University, Suzhou 215123, China; 20174214077@stu.suda.edu.cn (L.X.); 20124def10@gmail.com (J.L.); Yina.Liu@xjtlu.edu.cn (Y.L.)
4 Jiangsu Key Laboratory for Carbon-Based Functional Materials and Devices, Institute of Functional Nano and Soft Materials (FUNSOM), Soochow University, Suzhou 215123, China; jacklei_zjou@163.com
* Correspondence: wenzhen2011@suda.edu.cn (Z.W.); ylxie2013@sinano.ac.cn (Y.X.); xhsun@suda.edu.cn (X.S.)

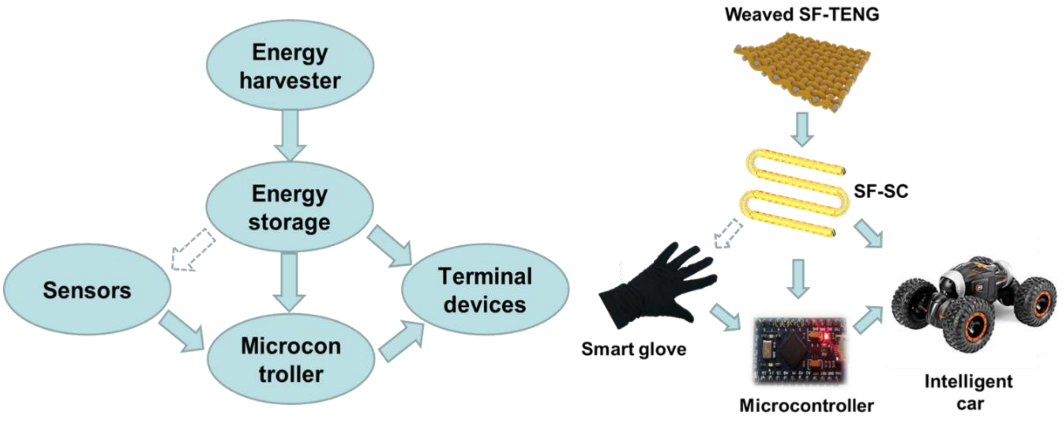


**Figure S1.** The working flow chart of an all-in-one self-powered human-machine interaction system.

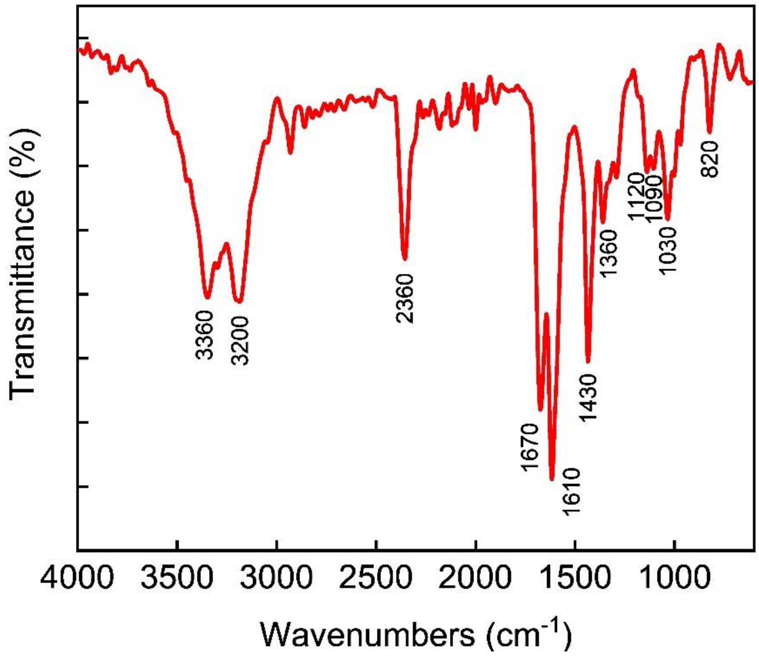


**Figure S2.** FTIR spectra of the freeze-dried composite hydrogel.

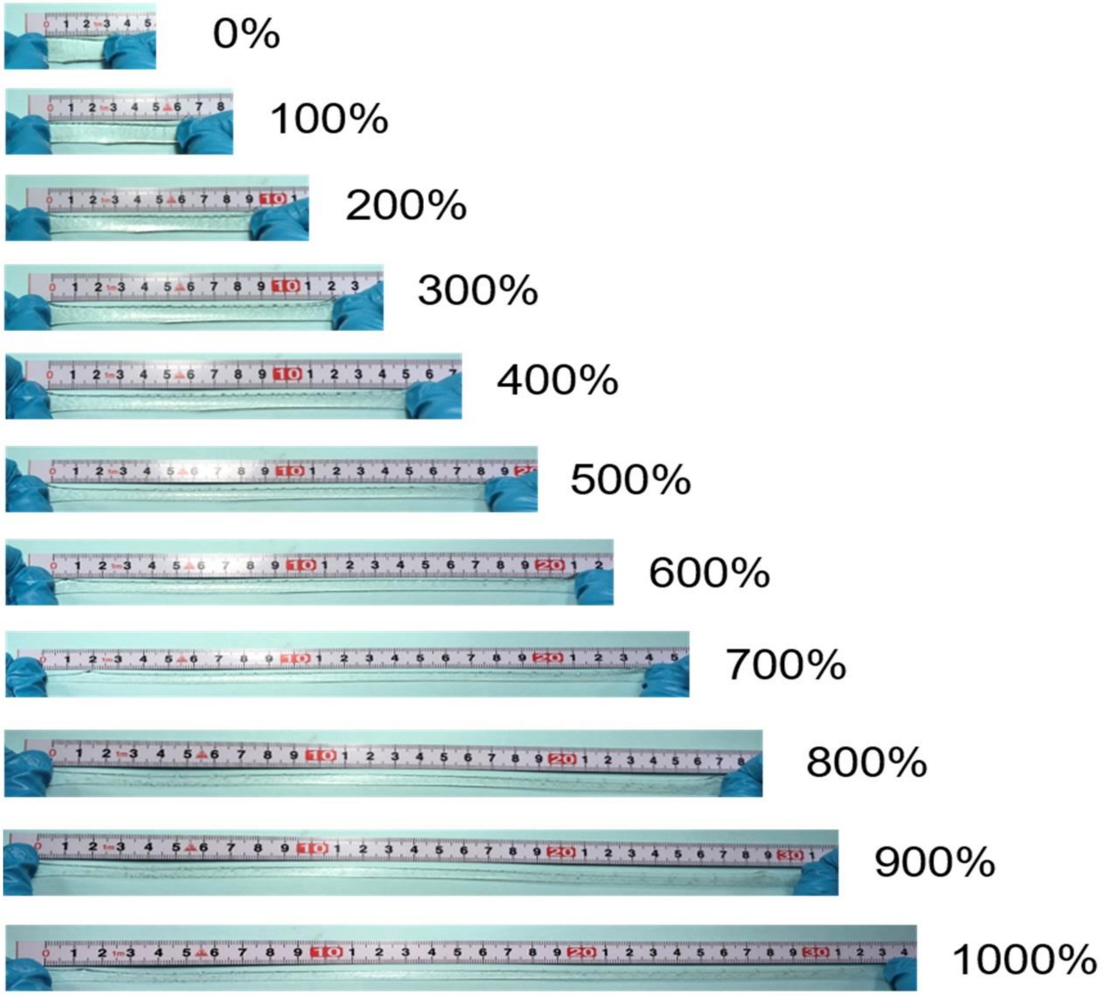


**Figure S3.** Optical images of hydrogel with different strains. The content of NaCl was fixed as 1.5g.

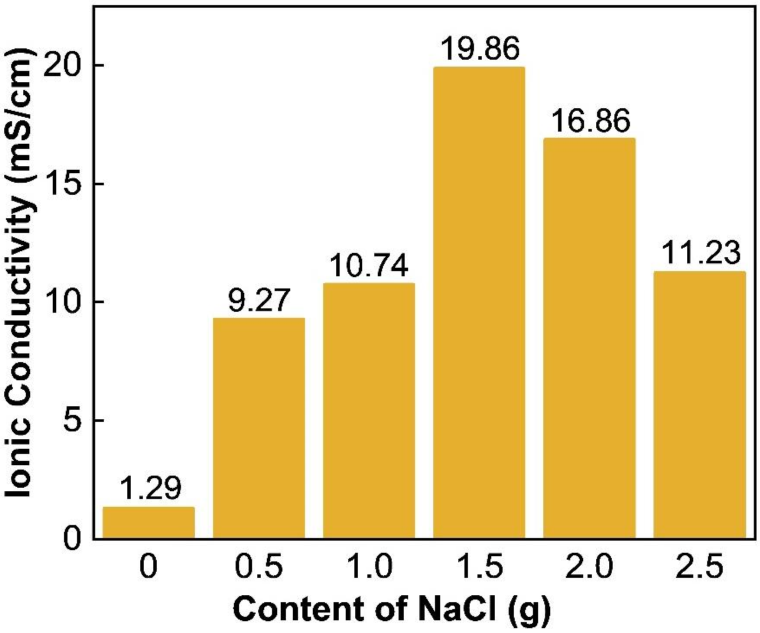


**Figure S4.** The calculated ionic conductivity(σ) of hydrogel with different NaCl contents. The maximal ionic conductivity is 19.86 mS/cm, which is 14 times higher than hydrogel without NaCl.

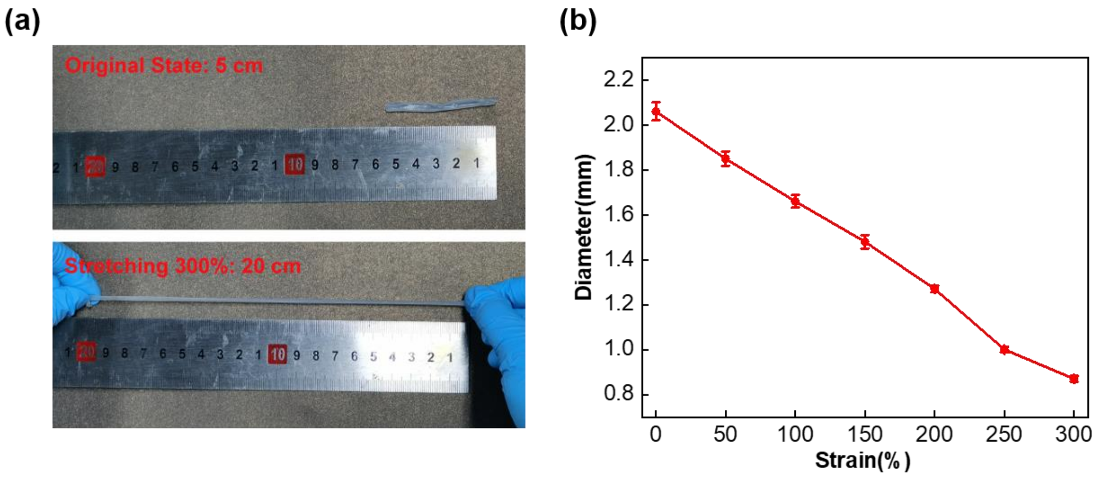


**Figure S5.** (**a**) Photos of SF-TENG at the original state and stretched state of 300% strain, (**b**) Diameter change at different stretch rates (0–300%).

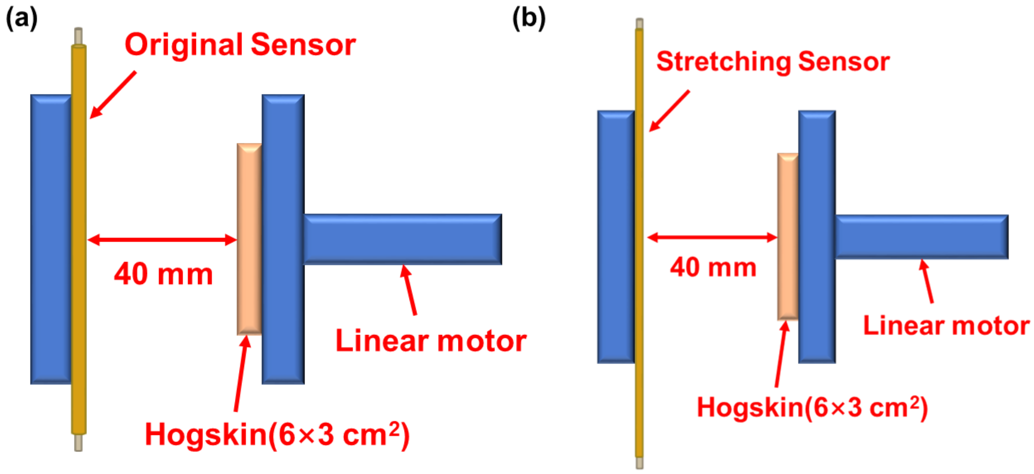


Figure S6. The testing schematic diagram of TENG at (**a**) original and (**b**) stretching state.

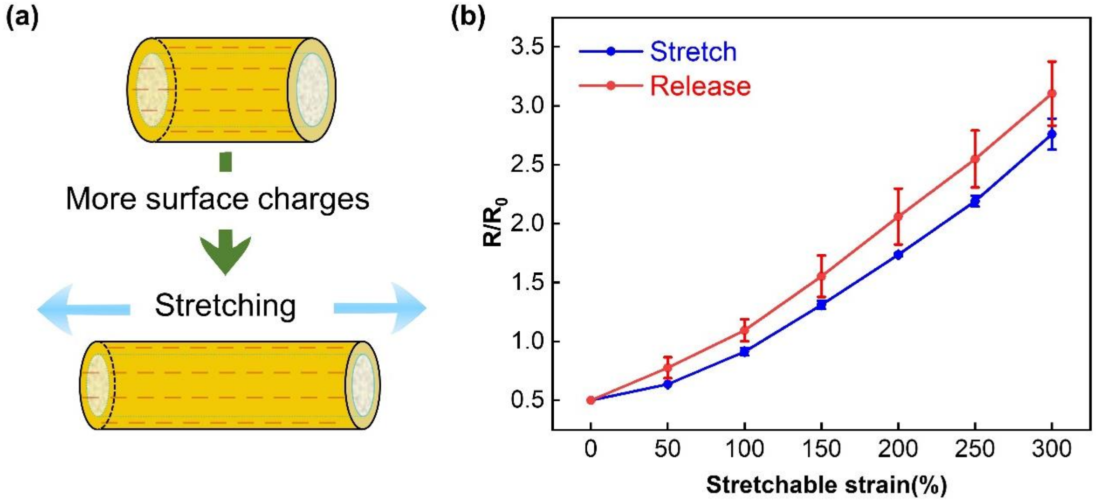


**Figure S7.** The surface of silicone rubber becomes thinner (**a**) and the resistance of hydrogel increase (**b**) during stretching.

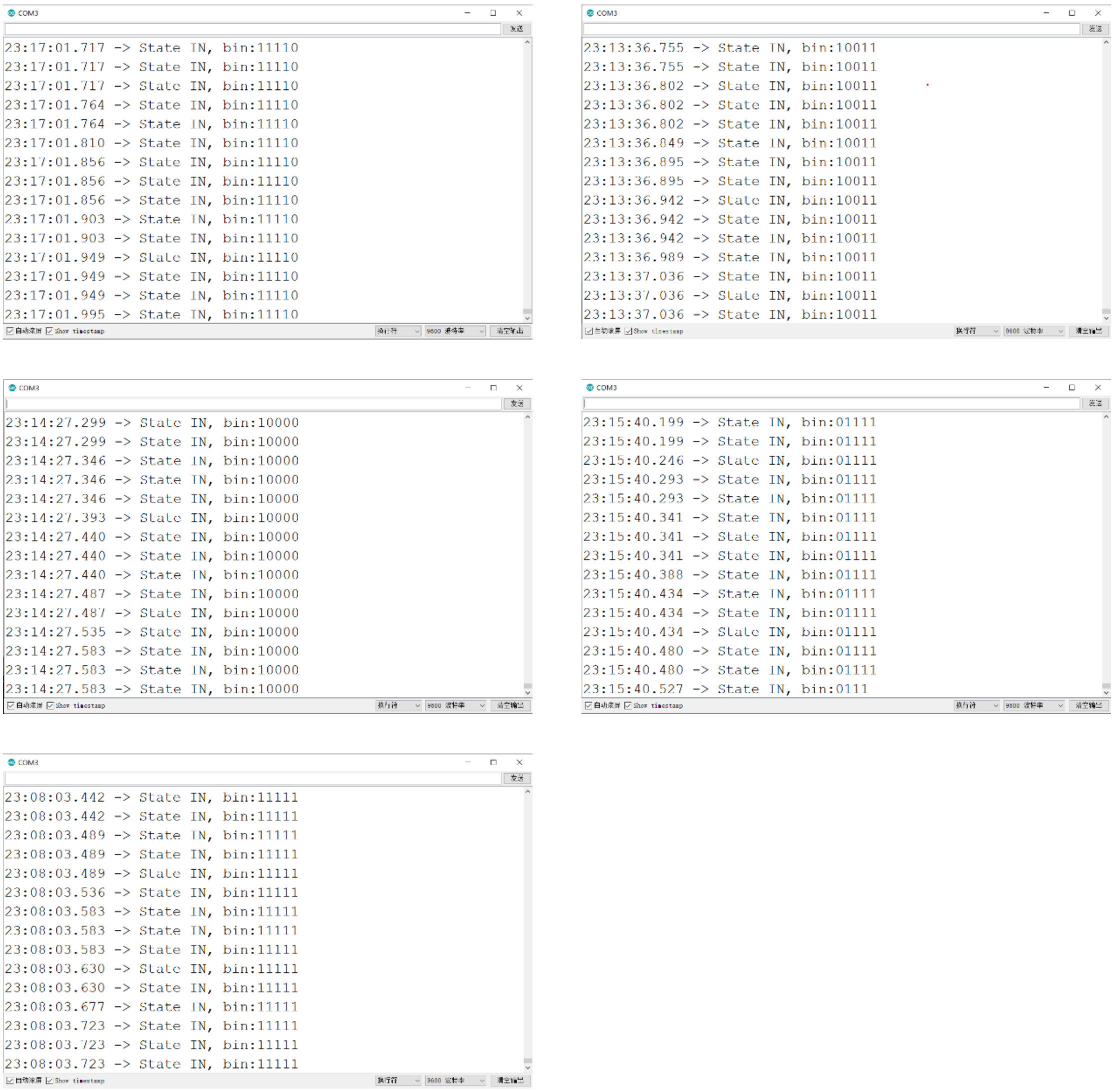


**Figure S8.** The processed and decoded RC command via a programmable mapping logic system.

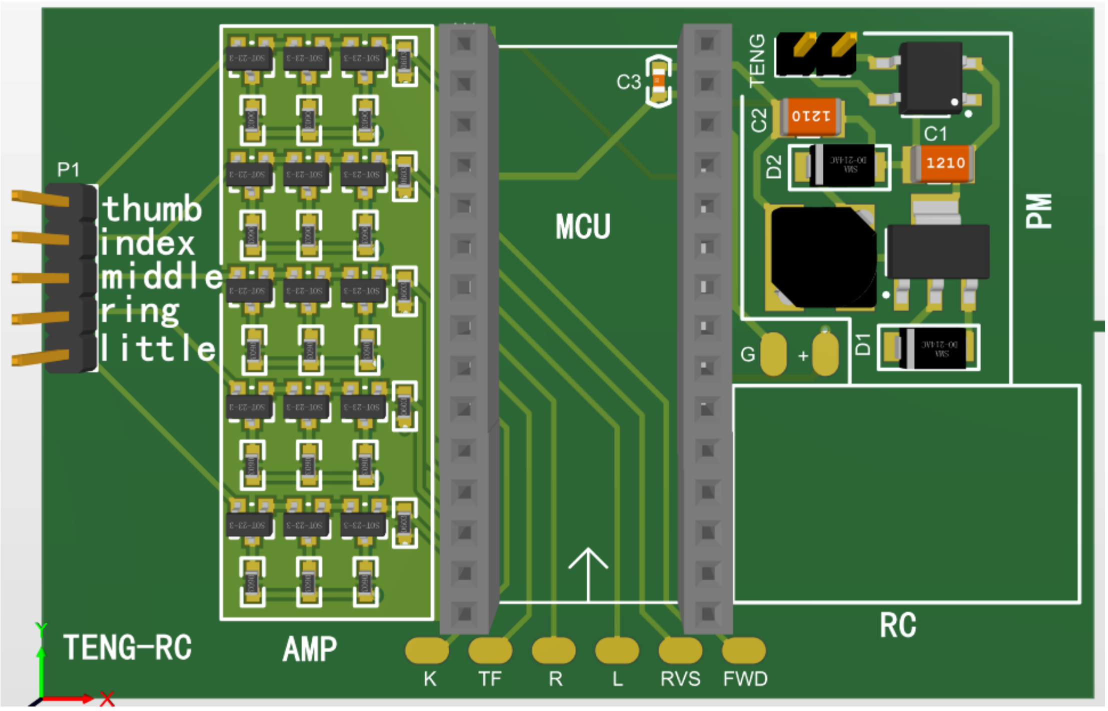


**Figure S9.** The circuit model for connecting the smart gloves and terminal devices.
